# Diagnostic Assessment of Tamoxifen-Associated Endometrial Pathology: A Narrative Review of Biomarkers, Risk Models, and Translational Gaps

**DOI:** 10.3390/diagnostics16203388

**Published:** 2026-10-18

**Authors:** Mohammed Ageeli Hakami

**Affiliations:** Department of Clinical Laboratory Sciences, College of Applied Medical Sciences, Shaqra University, Al-Quwayiyah, Riyadh 19257, Saudi Arabia; m.hakami@su.edu.sa

**Keywords:** tamoxifen, endometrial pathology, diagnostic accuracy, hysteroscopy, biomarkers, DNA methylation, risk prediction, narrative review

## Abstract

In tamoxifen-related endometrial alterations, a challenge arises due to the similarity between clinically significant diseases and both benign proliferation and benign polyps. This narrative review examines the clinical assessment, imaging, tissue sampling, biomarkers, and prediction models for women taking tamoxifen. We searched PubMed/MEDLINE and selected articles from reference lists up to August 2026, focusing on human studies reporting diagnostic performance, biomarkers, sampling limitations, or clinical prediction. Evidence was divided into three groups: tamoxifen-specific clinical studies, external studies of endometrial cancer, and mechanistic investigations; however, the heterogeneous populations and endpoints prevented pooled accuracy estimates. Although thickening of the endometrium is associated with an increased risk of abnormal bleeding, the finding when there is no abnormal bleeding is not significant enough to warrant routine surveillance of asymptomatic users. Representative histopathology remains the key, especially if hysteroscopy is used for sampling, particularly if the area is focal or if a diagnosis is not reached. These tissue markers primarily reflect the biology of exposure and show promise in methylation, cytology, serum-marker, and prediction studies, mostly in populations that are not tamoxifen-validated. Thus, reported discrimination does not provide sufficient reassurance to exclude atypical hyperplasia or carcinoma in tamoxifen users. The priorities are prospective validation in well-defined symptomatic patient groups, independent validation of locked assays or models against clinical baseline, and a focus on missed significant disease, avoided procedures, and patient outcomes. The current evidence favors a cautious diagnostic evaluation and does not define a biomarker-based screening or surveillance approach for women taking tamoxifen.

## 1. Introduction

Tamoxifen, a selective estrogen receptor modulator (SERM), is a key drug used in the treatment of estrogen receptor (ER)-positive breast cancer [1]. The endometrium has partial estrogenic activity and is involved in polyps, hyperplasia, and increased risk of endometrial carcinoma; however, these are not the same as changes that require treatment and are associated with exposure [2,3]. The diagnostic challenge is to differentiate between benign changes and the presence of atypical hyperplasia/endometrial intraepithelial neoplasia (EIN) or carcinoma without referring patients to invasive investigation.

The American College of Obstetricians and Gynecologists (ACOG) recommends that any abnormal vaginal bleeding, spotting, or bloody discharge in a tamoxifen user should be investigated, but only “routine” surveillance of the endometrium is indicated unless the patient is known to be at high risk [4]. Transvaginal ultrasonography (TVUS) can be used to characterize the uterus; however, tamoxifen-induced cystic and stromal changes make it difficult to interpret endometrial thickness [5]. Histopathology is crucial in the classification of a lesion, and the accuracy of a negative result is also dependent on the representativeness of the lesion sampled.

This review seeks to identify which results can be used in clinical assessment today, which ones are candidate investigational tests, and which evidence is needed before they can impact the biopsy decision-making process. It helps to distinguish between diagnostic triage for a symptomatic tamoxifen user, screening of asymptomatic tamoxifen users, and molecular characterization of a cancer already diagnosed. Data from general endometrial cancer cohorts are not viewed as measured performance in women receiving tamoxifen, but as potential approaches to be emulated in the future.

## 2. Literature Identification and Evidence Synthesis

### 2.1. Review Design and Literature Identification

This is a critical narrative review, rather than a systematic review, scoping review, or diagnostic accuracy meta-analysis. Initial literature identification was done using MEDLINE/PubMed literature search and backward reference search up to August 2026. Executable queries, lower date boundaries, language filters, and record-level screening logs of the initial literature identification are not available, and the identification, exclusion, and inclusion counts cannot be reconstructed reliably. A Preferred Reporting Items for Systematic Reviews and Meta-Analyses (PRISMA) flow diagram or its scoping-review extension (PRISMA-ScR) would suggest a defined exhaustive selection process, which was not performed and is not reported here.

The numerical claims and bibliographic records were verified using PubMed and available publisher reports. The search was expanded using PubMed in October 2026, using the same publication window from 2000, but with no lower publication restriction other than the search range. The query included tamoxifen along with any of the terms: endometrial/endometrium/uterine, diagnostic, imaging, sampling, biomarker, methylation, genomic, and prediction; the full executable query and the number of retrieved records are listed in Supplementary Material S1. This query records the extent of the search, and there was no systematic screening of the retrieved record set, nor should the number of retrieved records be regarded as the number of included studies.

Directly relevant evidence for this synthesis included human cohorts exposed to tamoxifen, focusing on the risk of endometrial disease, clinical assessment, sampling, diagnostic assessment, and prediction. External human endometrial cancer studies were retained if they assessed an assay or model that could be applied for potential future use in human diagnosis. Only human tissue studies and mechanistic parts of translational studies were used to explain the biological plausibility. There was no lower limit on the sample size, as small discovery studies were part of the evidence gap being addressed. Reporting was done in English or English abstracts were used; no systematic attempt was made to search or translate non-English literature. Reports of conference-only or breast cancer outcome studies not related to this study were not used to support the clinical diagnostic performance, nor were isolated cell culture findings.

### 2.2. Appraisal and Synthesis

The review was performed according to the six domains of the Scale for the Assessment of Narrative Review Articles (SANRA): importance, aims, search description, referencing, scientific reasoning, and presentation of relevant outcomes [6]. In Supplementary Material S1, these domains are mapped to the manuscript, and no self-rated quality score is provided. SANRA is used to guide the quality of narrative reviews and is not used to assess bias in primary studies.

No formal Quality Assessment of Diagnostic Accuracy Studies 2 (QUADAS-2) or Prediction model Risk of Bias Assessment Tool (PROBAST) ratings were made [7,8]. A protocolized, study-design-specific full-text assessment with independent judgments was outside the scope of this targeted narrative review; therefore, the selected literature includes incidence cohorts, diagnostic studies with different target conditions, tissue-discovery experiments, and prediction studies. Failing that, concerns specific to each study regarding selection, reference standards, threshold, sample adequacy, validation, and applicability are identified in the tables and Supplementary Table S1. Most of these studies in this qualitative appraisal have not been formally assessed, and no study can be at low risk of bias.

Studies were analyzed based on exposure status, menopausal and symptom profile, recruitment setting, specimen, condition to be targeted, and validation design. Sensitivity, specificity, and area under the receiver operating characteristic curve (AUC) were only reported for the population and endpoint they were measured in, and confidence intervals were retained for key estimates. Pooled accuracy, cross-study ranking, and newly calculated clinical effect estimates were not presented. For instance, no test evaluating any endometrial abnormality could be presumed to have the same performance as excluding EIN and/or carcinoma.

## 3. Clinical Spectrum and Determinants of Endometrial Risk

### 3.1. Spectrum of Endometrial Pathology

The clinical spectrum includes inactive or atrophic endometrium, benign polyps, endometrial hyperplasia without atypia, EIN/atypical hyperplasia, and endometrial carcinoma. In a retrospective study of 700 tamoxifen-treated women, Deligdisch et al. reported that all patients were examined for bleeding and/or abnormal sonography; thus, the high prevalence of benign or non-pathological findings does not reflect the prevalence among all tamoxifen users [9]. Similarly, among the 85 patients referred for lesions, the ultrasound-referred and bleeding groups in the study by Toroń et al. were both clinically selected, showing that polyps were the most common lesion [10]. These studies demonstrate differential diagnosis, but not the yield of population screening.

A national cohort of 78,320 women with a diagnosis of breast cancer who were classified as premenopausal found that tamoxifen was linked to an increased incidence of uterine disease, including endometrial cancer, although claims-based assessment of exposure and classification as premenopausal, as well as subsequent changes in treatment and surveillance patterns, limit causal interpretation [11]. Older selected cancer series have reported adverse histological characteristics following tamoxifen exposure, but these characteristics cannot be used to calculate the risk of malignancy in an individual woman with benign-appearing thickening [12]. The clinically important target for most triage studies should be defined as EIN/atypical hyperplasia and/or carcinoma, and benign polyps and non-atypical hyperplasia should be reported separately.

### 3.2. Patient and Treatment Factors

The risk increase due to tamoxifen is not the same for all age groups, as baseline risks vary with age and menopausal status. Chu et al. reported that breast cancer patients treated with tamoxifen alone had a higher risk of endometrial cancer compared with nonusers in a population-based cohort of 39,216 women in Taiwan, with an adjusted hazard ratio (HR) of 3.74 (95% confidence interval [CI] 1.65–8.48) in women aged 40–49 years [13]. This does not imply that there is a consistent increase in the treatment-associated relative risk with advancing age, but that this is the case in that age stratum and setting.

**Figure 1:**
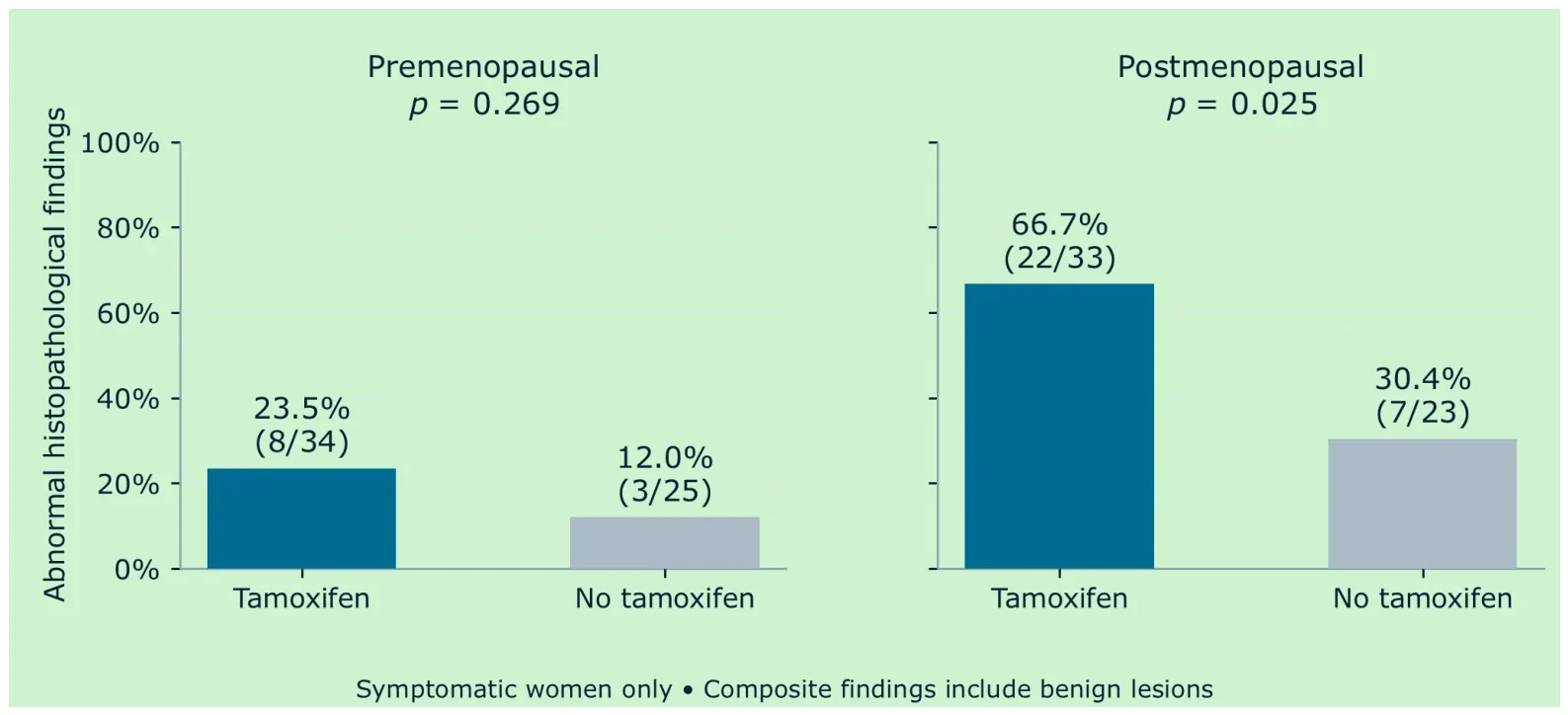
Endometrial pathological changes in symptomatic breast cancer cases according to menopausal status and tamoxifen use. Values and numerators/denominators are provided from Cheng et al. [14], and *p*-values are the within-stratum comparisons. The composite outcome is not the rate of endometrial cancer but includes benign abnormalities. This figure is a descriptive presentation of one of the prospective studies and is not a pooled effect, screening estimate, or formal test of interaction.

Cheng et al. prospectively compared a group of breast cancer patients who were exposed to tamoxifen with a group of breast cancer patients who were not exposed to tamoxifen. In premenopausal women, the proportions of women with abnormal histopathological findings were 8/34 (23.5%) and 3/25 (12.0%) for users and non-users of pills, respectively (*p* = 0.269); and 22/33 (66.7%) and 7/23 (30.4%) for users and non-users of pills, respectively, among postmenopausal women (*p* = 0.025) [14]. These reported proportions and denominators are shown in Figure 1. If the endpoint included benign abnormalities, all were symptomatic, and the result of the smaller premenopausal comparison was nonsignificant, this is not proof of equivalence or proof of a formal interaction with menopausal status.

The duration of exposure and other hormonal and metabolic risk factors are also important. Bernstein et al. conducted a nested case–control study involving women diagnosed with breast cancer. They discovered that using tamoxifen for over five years heightened the risk of endometrial cancer. This risk was influenced by prior estrogen replacement therapy and body mass. However, this finding does not serve as a diagnostic threshold for individual patients [15]. In another retrospective breast cancer cohort, Vizzotto et al. linked obesity and tamoxifen treatment to endometrial changes using a more liberal definition of outcome than cancer, namely endometrial changes [16]. These factors would be included as clinical assessment and validation-study cofactors but would not be a substitute for symptom evaluation or a representative tissue.

Similar caution is required for estimates of risk for referral-cohort groups. In a study of 249 women with endometrial samples treated with tamoxifen, hyperplasia (with or without atypia)/cancer was associated with nulliparity and endometrial thickness ≥ 9 mm, although only nine women had this combination [17]. Lee et al. observed that abnormal bleeding was related to hyperplasia or cancer in 284 biopsies of premenopausal women taking tamoxifen for breast cancer, but the low number of cancers restricted the accuracy of the test [18]. Neither study confirmed a universal thickness threshold or a validated screening schedule.

### 3.3. Symptomatic and Asymptomatic Clinical Settings

The group of women with abnormal uterine bleeding is a diagnostic population; the first question should be whether there is a clinically important lesion to account for the symptom. There are potential screening populations such as asymptomatic tamoxifen users, in which disease prevalence is lower; however, there are more frequent benign changes that can have a more negative impact if the results are false-positive. In the prospective postmenopausal tamoxifen surveillance, Gerber et al. reported that most of the ultrasound abnormalities reported were benign and that the procedural morbidity was low, which shows that more abnormalities detected do not equate to better results [19]. The longitudinal ultrasound surveillance by Fung et al. also resulted in numerous false-positive results and a low positive predictive value for cancer [20].

There is an extra source of attribution error due to pre-existing abnormalities. Lopez et al. analyzed asymptomatic postmenopausal women with estrogen receptor-positive breast cancer prior to treatment and identified a high baseline burden, with mostly benign pathology [21]. Findings by Garuti et al. revealed that both the time of exposure in pretreatment and follow-up observations, as well as baseline uterine status, should be documented in diagnostic studies [22]. These observations do not prove that all asymptomatic women should have baseline hysteroscopies, but rather that any abnormalities found following tamoxifen therapy cannot be solely blamed on the drug’s effect.

### 3.4. Population Boundaries and Interpretation of Reported Results

The evidence used in this review was categorized into three categories. Direct evidence comes from an exposed clinical cohort or an exposed subgroup that is separately reported, but it may only be applicable to symptomatic or biopsy-selected women. External clinical evidence refers to populations of patients with endometrial disease that have not been separately validated for tamoxifen use and are used to identify candidates for testing, not to estimate tamoxifen performance. Mechanistic evidence provides an explanation of tissue response or known tumors and can be used to support possible biological plausibility without showing detection before diagnosis. The boundaries and decisions that each category can make are summarized in Table 1.

## 4. Current Diagnostic and Laboratory Assessment

### 4.1. Clinical and Imaging Assessment

Inappropriate bleeding should first be evaluated by taking a bleeding history, menopausal status, tamoxifen exposure, other risk factors, and potential non-endometrial causes of bleeding. Franchi et al. found that bleeding and endometrial thickness >9 mm were related to abnormal endometrial findings in 163 of 185 postmenopausal women who were administered tamoxifen and selected for bleeding or for having an endometrium >4 mm thick [23]. These associations, based on referrals, can raise concerns about a lesion but do not validate not performing investigations below the threshold.

TVUS is an endpoint- and patient selection-dependent method. Weaver et al. observed high sensitivity and low specificity at a threshold of 5 mm in symptomatic postmenopausal women treated with tamoxifen (Table 2) [24]. In a separate group of women over 50 years of age, Liedman et al. demonstrated that apparent thickening may be related to atrophy, and distortion due to changes in the surrounding stromal or myometrial tissue is possible [25]. These findings account for the poor rule-in performance of thickness, and if bleeding persists or recurs, despite a reassuring initial result, this should be considered.

In addition, thresholds for menopause are exploratory. Huang et al. retrospectively examined 205 biopsies among tamoxifen-treated women and derived different thresholds and AUCs in the premenopausal and postmenopausal groups; however, the outcome assessed was more general, which included endometrial pathology, and the thresholds lacked independent validation [26]. With saline-infusion sonohysterography (SIS), there is enhanced specificity for delineation of focal intracavitary lesions (Fong et al. studied asymptomatic postmenopausal users; successful completion varied by procedure, and lesions were polyps or submucosal fibroids) [27]. Develioğlu and colleagues conducted a retrospective study of 60 asymptomatic users, showing that SIS was able to provide additional information, while Doppler could not provide similar diagnostic value [28]. These results were for anatomical characterization and not for the exclusion of EIN or carcinoma.

The utility of ultrasound as a gatekeeper can be limited in a symptomatic tamoxifen population. In a study of 614 women with postmenopausal bleeding, Abdaal et al. identified 98.1% of the 53 women taking or having previously taken tamoxifen as requiring additional hysteroscopy and biopsy [29]. Therefore, it should be compared with a well-defined symptom-led diagnostic pathway and should have good evidence that no significant disease is being misdiagnosed because of not performing the procedure.

**Table 2:** Selected diagnostic studies grouped by test type and applicability.

Test and Evidence	Population and Reference Standard	Target Outcome	Reported Result	Interpretive Limitation
TVUS, 5 mm threshold [24] Direct	Symptomatic postmenopausal tamoxifen users; histopathological assessment	Endometrial pathology	Sensitivity 97% (95% CI 83–100); specificity 35% (17–56)	Composite pathology; low specificity; not an asymptomatic screening estimate
TVUS thresholds [26] Direct	205 biopsies in tamoxifen users; separate menopausal groups; biopsy histology	Endometrial pathology	AUC 0.845 at 9.5 mm before menopause; 0.759 at 5.5 mm after menopause	Retrospective, biopsy-selected sample; thresholds derived within the cohort
TVUS and SIS [27] Direct	138 asymptomatic postmenopausal users; combined hysteroscopy–histopathology standard; 92 completed all procedures	Intracavitary abnormality, principally polyps	TVUS sensitivity/specificity 85.1%/55.7%; SIS 89.7%/79.2% in complete-procedure subgroup	Different evaluable denominators; benign structural endpoint; no cancer rule-out inference
Office biopsy [30] Direct	111 assessable tamoxifen users; 635 repeated biopsies	Adequacy of tissue for diagnosis	82/635 biopsies insufficient (12.9%)	Biopsy-level observations are correlated within women; inadequacy is not sensitivity
D&C followed by hysteroscopy [31] Direct	59 asymptomatic tamoxifen users with thickening; histology	Sampling failure and missed focal polyps	24/59 D&C samples unavailable; polyps found in 11/24 subsequent directed biopsies	Sequential investigation of failed samples; not a head-to-head cancer-accuracy study
Blind Novak biopsy [32] External	General referred gynecological cohort; hysteroscopic assessment	Endometrial polyps	Sensitivity 8.4%	Not tamoxifen-specific; focal-lesion outcome, not carcinoma exclusion
Serum HE4 and CA125 [33] External	755 women including cancer cases and controls; histology/final clinical classification	Endometrial cancer	Combined-marker AUC 0.77	No independently validated tamoxifen subgroup or clinical-utility evidence
Uterine-lavage sequencing [34] External	107 recruited for hysteroscopy/curettage; tissue histopathology	Cancer-associated mutations versus cancer diagnosis	Mutations detected in all 7 cancers and in 51 women without histopathological cancer	Limited cancer count; mutations also occur without a diagnosed malignant lesion
Three-marker methylation panels [35] External	103 known cancers; 317 unpaired healthy-control samples; histologically confirmed cases	Endometrial cancer	AUC 0.95 in urine, 0.94 in self-samples, 0.97 in cervical scrapes	Case–control spectrum; leave-one-out internal validation; no tamoxifen-specific benign controls
EMPap methylation classifier [36] External	1297 women with suspected cancer; 584 training and 713 validation; 196 cancers overall; pathology	Endometrial cancer	Overall AUC 0.93; sensitivity/specificity 90.3%/89.3%; validation cohort 87.9%/87.8%	Post-biopsy sampling and exclusion of discordant pathology affect interpretation; no tamoxifen-specific validation
Urine and vaginal cytology [37] External	103 known cancer cases and 113 women with unexplained postmenopausal bleeding; clinical/pathological diagnosis	Gynecological cancer detection	Sensitivity 91.7% (95% CI 85.0–96.1); specificity 88.8% (81.2–94.1)	Enriched cross-sectional cohort; broader cancer endpoint; no tamoxifen-specific validation

### 4.2. Endometrial Sampling and Histopathological Assessment

Histopathology is essential for pathognostic diagnosis, and hysteroscopy is used for better localization of focal lesions and for resolving inadequate or discordant sampling. Do not blame the limitations of blind sampling on directed sampling. Blind Novak curettage had poor sensitivity for the detection of polyps in the general gynecological cohort studied by Svirsky et al. and is an outside estimate of the accuracy of focal lesion sampling, not specific to tamoxifen [32]. Barakat et al. provided direct evidence for tamoxifen: 111 assessable women had 635 office biopsies, 82 of which were inadequate for diagnosis (12.9%) [30]. These are not the 635 patients, but the biopsy-level counts.

In the asymptomatic group of women with tamoxifen use studied by Korkmazer and colleagues, 59 women with a thickened endometrium had a D&C performed, and 24 of them did not get any material from the D&C; 11 of the 24 women had a hysteroscopic biopsy which found polyps in 11 of these women [31]. This is not a false-negative rate of cancer but rather an unresolved focal disease after failed sampling. A better performance of hysteroscopy-directed biopsy compared to blind biopsy methods was suggested by external evidence from a retrospective cohort of 2054 premenopausal women, but the comparison was not randomized or limited to tamoxifen users [38].

The results of the tissue must be viewed in the context of specimen adequacy, focality, persistence of symptoms, and concordance with imaging results. In studies for tissue markers, it is important to record the acquisition technique, the lesion targeted, the cellularity, how it was fixed, how it was stored, and the platform on which the assay was run, to avoid confusing the effects of sampling and processing for any biological discrimination. Morphological classification and measurement of biomarkers are complementary in the diagnostic process, and if an assay is performed on an unrepresentative tissue, it cannot compensate for the missed lesion.

### 4.3. Minimally Invasive Tests and Unmet Diagnostic Needs

**Figure 2:**
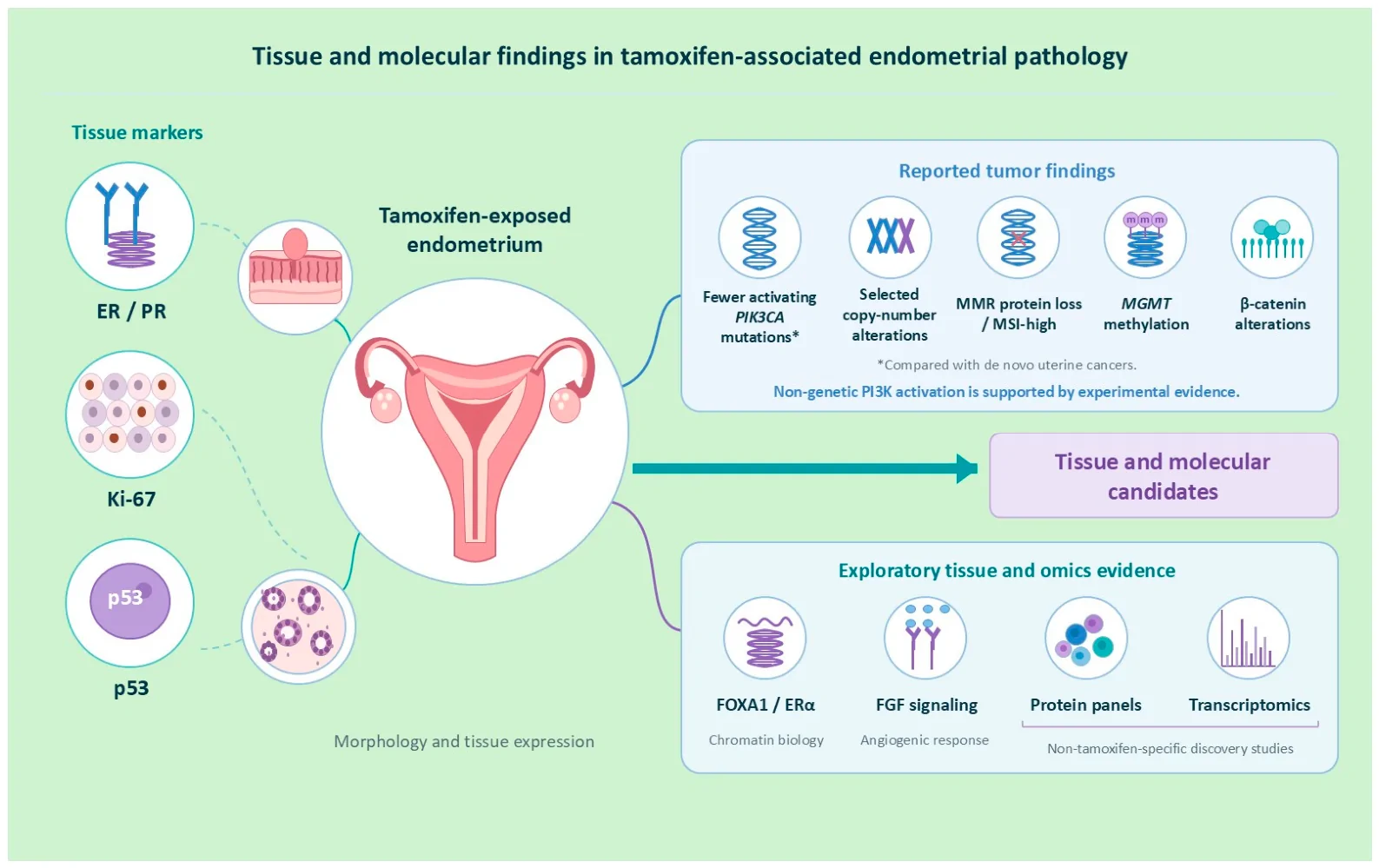
Current diagnostic assessment and investigational biomarker approaches in tamoxifen-associated endometrial pathology.

The most compelling translational rationale for a minimally invasive test is improvement in biopsy selection, while maintaining detection of EIN and carcinoma. However, the studies of serum, cytology, and methylation in Table 2 mostly included broad endometrial and/or gynecological cancer cohorts. Barr et al. compared the combined performance of serum human epididymis protein 4 (HE4) and cancer antigen 125 (CA125) in 755 women, but the combination of these two markers did not make a stand-alone test for tamoxifen-associated disease [33]. A model that incorporates clinical variables should also show incremental value over clinical variables. Figure 2 provides a summary of the current clinical evaluation and outlines the differentiating features between existing diagnostic methods and investigational biomarker methods that need to be validated in tamoxifen-exposed populations.

Various molecular signals pose various specificity issues. In a study of seven women with endometrial cancer, Nair et al. found cancer-associated mutations in uterine lavage of all the women, but also in 51 women without a histopathological diagnosis of cancer, demonstrating that mutations are insufficient to determine an actionable lesion [34]. Wever et al. assessed methylation panels in samples from 103 known endometrial cancer cases and unpaired healthy controls. However, strong discrimination in such a setting may be influenced by the case–control spectrum and does not define specificity between the various benign endometrial lesions related to tamoxifen use [35].

Ma et al. tested the EMPap methylation classifier on 1297 women who were suspected to have endometrial cancer, of whom 196 had confirmed endometrial cancer [37]. O’Flynn et al. merged known cases of cancer with women who were being evaluated for postmenopausal bleeding and reported on urine and vaginal cytology use for gynecological cancer detection [37]. Both published overall results are not validated tamoxifen-specific rule-out estimates, and the cytology endpoint is not specific to endometrial cancer. The next question is whether the assays can discriminate between stromal changes, tamoxifen-associated polyps, and EIN or carcinoma in subsequent patients who are subjected to the same work-up.

## 5. Tissue Biomarkers and Molecular Pathology

### 5.1. Hormonal and Proliferative Markers

Receptor or proliferation changes are not equivalent to markers of malignant potential in the case of exposure. Leão et al. compared PR and ER expression in 84 polyps from postmenopausal women exposed to tamoxifen with 252 polyps from postmenopausal women who were not exposed to tamoxifen, and 84 atrophic controls, and found that PR expression was higher in postmenopausal women who had been exposed to tamoxifen compared to those who were not, but ER expression was not different between the groups of polyps [39]. Therefore, Tregón et al. investigated 40 postmenopausal women awaiting surgery for prolapse (20 receiving tamoxifen for 21 days) and showed receptor and Ki-67 responses [40]. This was an experimental exposure study to provide insights into tissue biology, not long-term diagnostic performance in breast cancer survivors.

There are also disagreements regarding small polyp research. Altaner et al. reported a higher Ki-67 index in tamoxifen-associated polyps compared to controls, but no difference in ER, PR, Ki-67, or Bcl-2 between 14 tamoxifen users and 32 nonusers with postmenopausal bleeding and resected polyps was reported by Dibi et al. [41,42]. Sampling, scoring, and case mix differences may play a role, and neither study provides a validated cutoff point to distinguish exposed normal tissue from EIN or carcinoma. These limitations are summarized in Supplementary Table S1; a detailed breakdown of the limitations study by study is not presented here.

In the study by Pokharel et al., 50 tamoxifen-treated women were studied, and the sensitivity of serum p53 antibody and tissue p53 immunohistochemistry against histopathology was 50.0% and 57.1%, respectively, indicating only fair-to-good performance for stand-alone screening [43]. Negoiţă and Mihailovici reported the receptor and proliferation patterns of benign and malignant lesions in 20 selected specimens exposed to tamoxifen and presenting with symptoms but failed to validate a diagnostic classifier [44]. These detection claims are different from the use of p53 and mismatch-repair immunohistochemistry, which are well established in the molecular characterization of already diagnosed endometrial carcinoma [2].

### 5.2. Genomic and Epigenetic Findings

Tumor profiling is applied for the detection of mechanisms and classification when cancer has already occurred. Kübler et al. reported reduced frequencies of activating mutations in the PI3K pathway in tamoxifen-related uterine cancers and confirmed a non-genetic mechanism of PI3K activation using experimental models [45]. Therefore, reduction in mutation frequency cannot be called a reduction in pathway activity and is not a validated early-detection signature. Saeki et al. performed a comparison between 10 ta-ETs and 20 sporadic ETs and identified selected copy number alterations and four tumors with MSI-high and concordant mismatch-repair protein loss [46]. The small exposed group and lack of an adequate benign exposed comparison prevent the calculation of a clinically useful triage threshold.

Other reported differences also generate hypotheses. Nagy et al. reported comparisons of 30 exposed and 38 unexposed cancers, where they found differences in both the prevalence of MGMT methylation and MGMT methylation–mutation co-occurrence (78% versus 50%) [47]. Turbiner et al. observed abnormalities in β-catenin but not in KRAS, PTEN, or microsatellite instability in small informative subsets of tamoxifen-associated cancers, which did not reach statistical significance [48]. These studies are not mutually consistent for the purpose of constructing a single molecular signature, as the assays, tumor histology, denominator, and comparator groups vary.

### 5.3. Transcriptomic and Proteomic Evidence

Omics observations are still mainly tissue-based. However, the association of ERα/FOXA1 expression with the interval between breast cancer and uterine cancer in tamoxifen-treated patients was not a diagnostic accuracy result [49], which was combined with chromatin studies in 230 primary tumors by Droog and colleagues. Hague et al. noted angiogenic responses, and these were noted as a response to treatment rather than the specific detection of malignant disease in the exposed pre-and PM endometrium [50].

**Figure 3:**
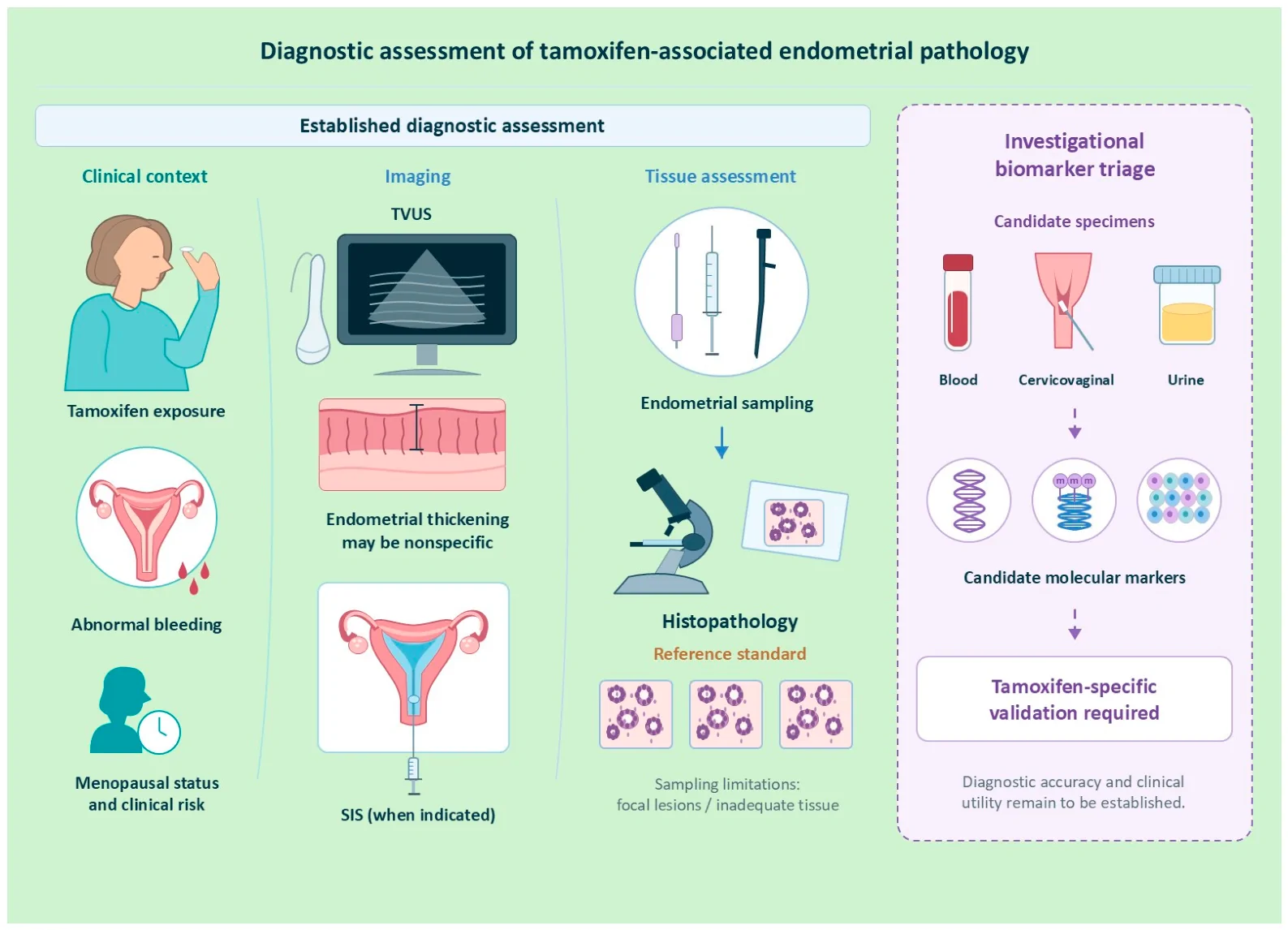
Tissue biomarkers and molecular findings in tamoxifen-associated endometrial pathology.

The translation gap is further illustrated in external discovery studies. Dubé et al. studied a 3-protein panel in a 148-sample tissue microarray, and Jin et al. sequenced RNA in only five paired cancer and normal tissues and performed tissue-level validation [51,52]. Both studies fall short of providing a specific tamoxifen biofluid performance. The validation of any minimally invasive diagnostic claim for discovery in resected tissue should include a verification of the discovery in the intended specimen and testing against a benign exposed control and independent validation at a prespecified threshold. As summarized in Figure 3, the aforementioned tissue markers and molecular findings are divided between biological markers and those that need confirmatory diagnostics.

## 6. Prediction Models and Clinical Translation

### 6.1. Clinical Prediction and Artificial Intelligence

The performance of a model is related to the desired outcome and validation setup, not the name of the method (whether it is called artificial intelligence or not). Cao et al. developed a model in 224 women who were premenopausal at the time of breast cancer diagnosis and who underwent diagnostic curettage following tamoxifen therapy due to abnormalities or symptoms [53]. The reported C-index was 0.874, and 0.014 was the mean absolute error associated with the calibration curve (called mean absolute error of conformity in the source). This is not a calibration slope or Brier score and should not be numerically compared with either. The model was developed using an internal split and bootstrap validation and does not create a rule-out tool for cancer.

Also included in the Cao report, but not reproduced here, is an internally inconsistent confidence interval for its training AUC, the confidence interval not containing the point estimate. However, due to reporting problems, the small size of the internal test sample and recruitment after selection for curettage limit interpretation [53]. Before clinical deployment, external validation is needed in consecutive tamoxifen users with a given target condition.

Table 3 compares the exposed-cohort model with three external models. Li et al. evaluated 858 postmenopausal women selected for histopathology and, by an inbuilt random split, predicted atypical hyperplasia or cancer [54]. Xu et al. adopted the retrospective case–control model with the metabolically focused model, and the high AUCs reported need to be evaluated for spectrum effects and replicated [55]. Ai et al. examined 550 postmenopausal patients with thickened endometria, of whom 385 were earlier patients and 165 were later patients from the same center, for development and validation, respectively [56]. While this is described as external validation in the source, it is temporal validation, as it was performed in one center, not independent geographic validation, and the later validation sample in the Xu study was also from the same center.

Note that AUC is a measure of discrimination, calibration is a measure of agreement between the predicted and observed risk, whereas the Brier score captures the overall error in probability prediction and should not be considered interchangeable with the other two measures. Similarly, decision-curve analysis is based on assumptions and thresholds for potential net benefit and does not demonstrate that a model will actually lead to reduced procedures or better outcomes in practice. Future model reports should include reporting of the recruitment pathway, how missing data were handled, how features were selected, how outcomes were defined, calibration assessment, and independent validation, following Transparent Reporting of a multivariable prediction model for Individual Prognosis Or Diagnosis (TRIPOD) guidance extended to artificial intelligence (TRIPOD+AI) [57].

### 6.2. Analytical and Clinical Validation Requirements

The first step to constructing a useful validation study is to state the decision to be changed. This may be screening (for the asymptomatic) versus selection (for the symptomatic) for hysteroscopy and would need evidence of benefit and harm. Benign tamoxifen-exposed controls are crucial, as a marker that distinguishes established cancer from healthy unexposed tissue might just indicate tamoxifen-induced proliferation. When designing future diagnostic studies, the index-test threshold, blinded interpretation, reference standard, timing, and patient flow should be reported according to the Standards for Reporting Diagnostic Accuracy Studies (STARD) principles [58].

Clinical threshold selection should be performed after analytical validation. Relevant requirements include specimen stability, limits of detection, precision near the decision threshold, batch effects, assay interference, and interlaboratory reproducibility. Tissue assays also require standardized fixation, lesional cellularity, and scoring; serum-marker models must include clinically relevant nonmalignant determinants of analyte levels. Candidate tests should be compared to a predefined base model that includes symptoms, menopausal status, age, exposure duration, and imaging characteristics that are known to be relevant to demonstrate added value.

These requirements apply to common laboratory indices. Sarioglu et al. reviewed the pathological results of 361 tamoxifen users who underwent biopsy and correlated pathological results with red-cell and platelet distribution widths, the majority of which were benign polyps [59]. The low frequency and wide range of results of these associations do not support an inexpensive cancer detection test. Instead of excluding indeterminate assays and poor reference samples from the principal performance analysis, prospective studies should report the disposition of such assays.

### 6.3. Clinical Utility and Prioritized Research

The most significant gaps would be filled by three linked studies. A prospective consecutive series of symptomatic tamoxifen users should document menopausal status and exposure prior to testing, distinguish between benign lesions and EIN/carcinoma, and have a blinded histopathological adjudication process with clear management of inadequate samples and follow-up of unresolved cases. This would create a good clinical baseline and minimize the selection issues that are prevalent in the literature.

Second, testing of one locked candidate assay/integrated model should be performed in an independent multicenter cohort with representative benign tamoxifen-associated pathology. It is important to predetermine the decision, threshold, acceptable missed rate of disease, subgroup analyses, and follow-up for a negative result in the protocol. The most favorable post hoc AUC should not be reported, but rather calibration, sensitivity for EIN/carcinoma, and added value over the baseline pathway.

Third, a prospective comparative impact study to compare whether the use of assays can decrease the number of unindicated hysteroscopies or biopsies without delaying the diagnosis of significant lesions should be performed after validation. The outcomes should encompass missed EIN/carcinoma, diagnostic delay, repeat sampling, procedural harm, patient experience, and cost. Screening for asymptomatic individuals requires a distinct evaluation of benefits and harms. The success observed in symptomatic populations is not anticipated to directly apply to asymptomatic populations.

### 6.4. Strengths and Limitations of the Review

The key strength is the focus on decision—a separation between direct evidence of exposure and external diagnostic and mechanistic findings, and a distinction between the detection of cancer and other lesion endpoints. The tables show recruitment settings, reference standards, validation designs, and important considerations regarding applicability, including sampling numbers, marker interpretation, and prediction metrics. These differences allow readers to determine how much an apparently positive finding is or is not relevant to the clinical question.

The review is constrained by the non-exhaustive selection of literature, incomplete documentation of the initial literature search, the use of only one main bibliographic database, and the use of available English-language literature. Duplicate independent screening, protocol registration, study-level risk-of-bias scoring, and quantitative synthesis were not documented in the review. Most of the direct diagnostic evidence is from small or selected referral groups, and some supporting molecular evidence is from known tumors, not from women who are being initially assessed. The conclusions offer substantiated clinical principles and research priorities. However, they do not establish the superiority of comparative tests, universal thresholds, or the effectiveness of biomarker-led surveillance.

## 7. Conclusions and Future Perspectives

When assessing an abnormality found on tamoxifen, it is important to distinguish between the investigation of a symptom and the characterization of an incidental finding and screening an asymptomatic population. Bleeding should be considered for assessment; endometrial thickness is not a perfect discriminator, and when a lesion needs to be classified, representative histopathology should be undertaken when bleeding occurs. There is no validated tissue, molecular, or predictive study that supports a biomarker-driven strategy to exclude EIN or carcinoma in tamoxifen users.

The key evidence gap is applicability: good results in normal cancer cohorts or in resected tissue cannot be transferred to women who have benign changes associated with tamoxifen. The performance of any endometrial lesion cannot be reclassified as performance for clinically significant disease. This heterogeneity suggests caution in making general statements about diagnostic accuracy and attests to the need for circumscribed claims in the clinical context.

The next priorities are a potential cohort of patients who are at risk to receive symptomatic tamoxifen with standardized histological endpoints, a locked assay or model compared to a prespecified clinical baseline, and a comparative study measuring avoided procedures and missed or delayed EIN/carcinoma. These actions would establish whether an integrated approach would assist in diagnosis and patient outcomes. However, candidate biomarkers should not be considered for routine screening of asymptomatic users until further studies are conducted.

## Figures and Tables

**Table 1:** Population and intended-use boundaries for interpreting the evidence.

Population or Evidence Category	Clinical Question	What the Evidence can Support	What it Cannot Establish
Symptomatic tamoxifen users	Diagnostic assessment of bleeding	Associations and test performance in the stated symptomatic or referred cohort	Accuracy or benefit of screening asymptomatic users
Asymptomatic tamoxifen users	Screening or evaluation of incidental findings	False-positive burden and lesion spectrum in the sampled setting	Benefit of routine surveillance without outcome studies
General endometrial disease cohorts	External diagnostic or model development	Candidate assays, predictors, and validation methods	Tamoxifen-specific sensitivity, calibration, or safety
Resected tumors, benign tissue, or experimental exposure	Biological characterization	Pathways and candidate tissue markers	Validated minimally invasive early detection or future cancer prediction

**Table 3:** Selected prediction models and the limits of transfer to tamoxifen users.

Model and Applicability	Population and Outcome	Discrimination Reported	Calibration or Validation Reported	Main Interpretation
Cao et al. [53] Direct	224 selected tamoxifen-treated women; premenopausal at breast cancer diagnosis; composite endometrial lesions	C-index 0.874; internal test AUC 0.891	Internal split and 1000 bootstrap resamples; calibration-curve mean absolute error 0.014	No independent external validation; composite lesions include more than EIN/cancer; printed training AUC CI inconsistent
Li et al. [54] External	858 postmenopausal women undergoing histology; atypical hyperplasia or cancer	Internal validation neural-network AUC 0.840 (95% CI 0.770–0.909)	70:30 random split; Brier score 0.099	Single-center internal validation; no separate tamoxifen assessment
Xu et al. [55] External	445 cancer cases and 299 healthy controls in total; 416 training, 179 internal validation, 149 later validation	Training AUC 0.984; internal 0.987; later validation 0.964	Internal validation plus a later-period cohort from the same institution	Healthy-control spectrum; temporal rather than geographic validation; no tamoxifen-specific assessment
Ai et al. [56] External	550 postmenopausal women with thickened endometrium; 385 development and 165 later validation; cancer endpoint	Training AUC 0.897; reported validation AUC 0.941	Calibration plots; validation in a later period at the same center	Temporal validation is not independent multicenter validation; no tamoxifen-specific calibration

## Data Availability

No new data were created or analyzed in this study. Data sharing is not applicable to this article.

## Supplementary Materials

The following supporting information can be downloaded at: https://www.mdpi.com/article/10.3390/diagnostics16203388/s1, Supplementary Material S1: search documentation and SANRA domain mapping; Supplementary Table S1: Qualitative appraisal of selected tissue and molecular studies [6,7,8,39,40,41,42,43,44,45,46,47,48,49,50,51,52,55,56].

## References

[1] Dong Y., Shao L., Li Q., Qi Y., Liu T., Wei S., Qi H. (2026). Cutting-edge advances in endocrine therapy for breast cancer (Review). Oncol. Lett..

[2] Urban R., Castellano T., Doll K., DeLair D., Wright J., Eskander R., Westin S., Powell M., Craig A., Growdon W. (2026). Endometrial cancer: A Society of Gynecologic Oncology evidence-based review and recommendations: Part I. Gynecol. Oncol..

[3] Ghanavati M., Khorshidi Y., Shadnoush M., Akbari M.E., Ardehali S.H., Chavarri-Guerra Y., Akbari A., Barragan-Carrillo R., Amin Amlashi M., Javid Z. (2023). Tamoxifen use and risk of endometrial cancer in breast cancer patients: A systematic review and dose-response meta-analysis. Cancer Rep..

[4] American College of Obstetricians and Gynecologists (2014). Committee Opinion No. 601: Tamoxifen and uterine cancer. Obstet. Gynecol..

[5] Van den Bosch T. (2012). Ultrasound in the diagnosis of endometrial and intracavitary pathology: An update. Australas. J. Ultrasound Med..

[6] Baethge C., Goldbeck-Wood S., Mertens S. (2019). SANRA-a scale for the quality assessment of narrative review articles. Res. Integr. Peer Rev..

[7] Whiting P.F., Rutjes A.W., Westwood M.E., Mallett S., Deeks J.J., Reitsma J.B., Leeflang M.M., Sterne J.A., Bossuyt P.M., QUADAS-2 Group (2011). QUADAS-2: A revised tool for the quality assessment of diagnostic accuracy studies. Ann. Intern. Med..

[8] Wolff R.F., Moons K.G.M., Riley R.D., Whiting P.F., Westwood M., Collins G.S., Reitsma J.B., Kleijnen J., Mallett S., PROBAST Group (2019). PROBAST: A Tool to Assess the Risk of Bias and Applicability of Prediction Model Studies. Ann. Intern. Med..

[9] Deligdisch L., Kalir T., Cohen C.J., de Latour M., Le Bouedec G., Penault-Llorca F. (2000). Endometrial histopathology in 700 patients treated with tamoxifen for breast cancer. Gynecol. Oncol..

[10] Toroń M., Wołoszyn A., Zielińska A., Kociszewska A. (2026). Analysis of histopathological results of the endometrium in breast cancer patients treated with tamoxifen. Preliminary report. Wiad. Lek..

[11] Ryu K.J., Kim M.S., Lee J.Y., Nam S., Jeong H.G., Kim T., Park H. (2022). Risk of Endometrial Polyps, Hyperplasia, Carcinoma, and Uterine Cancer After Tamoxifen Treatment in Premenopausal Women with Breast Cancer. JAMA Netw. Open.

[12] Magriples U., Naftolin F., Schwartz P.E., Carcangiu M.L. (1993). High-grade endometrial carcinoma in tamoxifen-treated breast cancer patients. J. Clin. Oncol..

[13] Chu S.C., Hsieh C.J., Wang T.F., Hong M.K., Chu T.Y. (2020). Younger tamoxifen-treated breast cancer patients also had higher risk of endometrial cancer and the risk could be reduced by sequenced aromatase inhibitor use: A population-based study in Taiwan. Tzu Chi Med. J..

[14] Cheng W.F., Lin H.H., Torng P.L., Huang S.C. (1997). Comparison of endometrial changes among symptomatic tamoxifen-treated and nontreated premenopausal and postmenopausal breast cancer patients. Gynecol. Oncol..

[15] Bernstein L., Deapen D., Cerhan J.R., Schwartz S.M., Liff J., McGann-Maloney E., Perlman J.A., Ford L. (1999). Tamoxifen therapy for breast cancer and endometrial cancer risk. J. Natl. Cancer Inst..

[16] Vizzotto A.O., Nicolau S.M., Lopes G.M., Castelo Filho A. (2023). Risk factors for the development of endometrial lesions in breast cancer patients using tamoxyphen: A retrospective cohort study. Rev. Col. Bras. Cir..

[17] Shum M.J.M. (2022). Risk factors for endometrial pathology in women with breast cancer and tamoxifen treatment. Hong Kong J. Gynaecol. Obstet. Midwifery.

[18] Lee M., Piao J., Jeon M.J. (2020). Risk Factors Associated with Endometrial Pathology in Premenopausal Breast Cancer Patients Treated with Tamoxifen. Yonsei Med. J..

[19] Gerber B., Krause A., Müller H., Reimer T., Külz T., Makovitzky J., Kundt G., Friese K. (2000). Effects of adjuvant tamoxifen on the endometrium in postmenopausal women with breast cancer: A prospective long-term study using transvaginal ultrasound. J. Clin. Oncol..

[20] Fung M.F., Reid A., Faught W., Le T., Chenier C., Verma S., Brydon E., Fung K.F. (2003). Prospective longitudinal study of ultrasound screening for endometrial abnormalities in women with breast cancer receiving tamoxifen. Gynecol. Oncol..

[21] López D.M., Fernández Y.G., Sánchez A.V., Alvarez M.I., Reynaldo M.I., Delgado R.C. (2013). Baseline hysteroscopic assessment of endometrium in asymptomatic postmenopausal women with estrogen receptor-positive breast cancer. Menopause.

[22] Garuti G., Grossi F., Centinaio G., Sita G., Nalli G., Luerti M. (2007). Pretreatment and prospective assessment of endometrium in menopausal women taking tamoxifen for breast cancer. Eur. J. Obstet. Gynecol. Reprod. Biol..

[23] Franchi M., Ghezzi F., Donadello N., Zanaboni F., Beretta P., Bolis P. (1999). Endometrial thickness in tamoxifen-treated patients: An independent predictor of endometrial disease. Obstet. Gynecol..

[24] Weaver J., McHugo J.M., Clark T.J. (2005). Accuracy of transvaginal ultrasound in diagnosing endometrial pathology in women with post-menopausal bleeding on tamoxifen. Br. J. Radiol..

[25] Liedman R., Lindahl B., Andolf E., Willen R., Ingvar C., Ranstam J. (2000). Disaccordance between estimation of endometrial thickness as measured by transvaginal ultrasound compared with hysteroscopy and directed biopsy in breast cancer patients treated with tamoxifen. Anticancer. Res..

[26] Huang L., Lu Y., Chen Y., Liu H., An J. (2025). Determining a Treatment Threshold Value for Endometrial Thickness in Women with Breast Cancer Receiving Tamoxifen. Int. J. Womens Health.

[27] Fong K., Kung R., Lytwyn A., Trudeau M., Chapman W., Nugent P., Glanc P., Manchul L., Szabunio D., Myhr T. (2001). Endometrial evaluation with transvaginal US and hysterosonography in asymptomatic postmenopausal women with breast cancer receiving tamoxifen. Radiology.

[28] Develioğlu O.H., Omak M., Bilgin T., Esmer A., Tüfekçi M. (2004). The endometrium in asymptomatic breast cancer patients on tamoxifen: Value of transvaginal ultrasonography with saline infusion and Doppler flow. Gynecol. Oncol..

[29] Abdaal A., Mushtaq Y., Khasati L., Moneim J., Khan F., Ahmed H., Bolton H. (2018). Post-menopausal bleeding—Is transvaginal ultrasound a useful first-line investigation in tamoxifen users?. Post Reprod. Health.

[30] Barakat R.R., Gilewski T.A., Almadrones L., Saigo P.E., Venkatraman E., Hudis C., Hoskins W.J. (2000). Effect of adjuvant tamoxifen on the endometrium in women with breast cancer: A prospective study using office endometrial biopsy. J. Clin. Oncol..

[31] Korkmazer E., Solak N., Tokgöz V.Y. (2014). Assessment of thickened endometrium in tamoxifen therapy. Turk. J. Obstet. Gynecol..

[32] Svirsky R., Smorgick N., Rozowski U., Sagiv R., Feingold M., Halperin R., Pansky M. (2008). Can we rely on blind endometrial biopsy for detection of focal intrauterine pathology?. Am. J. Obstet. Gynecol..

[33] Barr C.E., Njoku K., Jones E.R., Crosbie E.J. (2022). Serum CA125 and HE4 as Biomarkers for the Detection of Endometrial Cancer and Associated High-Risk Features. Diagnostics.

[34] Nair N., Camacho-Vanegas O., Rykunov D., Dashkoff M., Camacho S.C., Schumacher C.A., Irish J.C., Harkins T.T., Freeman E., Garcia I. (2016). Genomic Analysis of Uterine Lavage Fluid Detects Early Endometrial Cancers and Reveals a Prevalent Landscape of Driver Mutations in Women without Histopathologic Evidence of Cancer: A Prospective Cross-Sectional Study. PLoS Med..

[35] Wever B.M.M., van den Helder R., van Splunter A.P., van Gent M.D.J.M., Kasius J.C., Trum J.W., Verhoeve H.R., van Baal W.M., Hulbert A., Verhoef L. (2023). DNA methylation testing for endometrial cancer detection in urine, cervicovaginal self-samples and cervical scrapes. Int. J. Cancer.

[36] Ma X., Chen X., Liang J., Zhang J., Wu Q., Wang D., Huang X., Zi D., Chen D., Wan H. (2024). A Multicenter Cohort Study on DNA Methylation for Endometrial Cancer Detection in Cervical Scrapings. Cancer Med..

[37] O’Flynn H., Ryan N.A.J., Narine N., Shelton D., Rana D., Crosbie E.J. (2021). Diagnostic accuracy of cytology for the detection of endometrial cancer in urine and vaginal samples. Nat. Commun..

[38] Öztürk F., Sağnıç S., Fırtına Tuncer S., Tuncer H.A. (2025). Diagnostic Efficiency of Endometrial Sampling Methods and Risk Factors for Endometrial Carcinoma and Precursor Lesions in Premenopausal Women. J. Clin. Med..

[39] Leão R.B., Andrade L., Vassalo J., Antunes A., Pinto-Neto A., Costa-Paiva L. (2013). Differences in estrogen and progesterone receptor expression in endometrial polyps and atrophic endometrium of postmenopausal women with and without exposure to tamoxifen. Mol. Clin. Oncol..

[40] Tregón M.L., Blümel J.E., Tarín J.J., Cano A. (2003). The early response of the postmenopausal endometrium to tamoxifen: Expression of estrogen receptors, progesterone receptors, and Ki-67 antigen. Menopause.

[41] Altaner S., Gucer F., Tokatli F., Guresci S., Ozdemir C., Puyan F.O., Kutlu K. (2006). Expression of Bcl-2 and Ki-67 in tamoxifen-associated endometrial polyps: Comparison with postmenopausal polyps. Onkologie.

[42] Dibi R.P., Zettler C.G., Vanin C.M.M., Ribeiro R.V.P., Oliveira J.M., Kremer T.G., Borges J., Kato S.K. (2020). Immunohistochemical assessment of symptomatic postmenopausal endometrial polyps in tamoxifen users and nonusers: A case control study. Sao Paulo Med. J..

[43] Pokharel H.P., Bhatla N., Kriplani A., Mukhopadhyay A., Srivastava A., Ralhan R. (2006). Evaluation of endometrial changes and p53 expression in tamoxifen treated women: Comparison of various methods. Kathmandu Univ. Med. J. (KUMJ).

[44] Negoiţă M., Mihailovici M.S. (2011). Expression of hormonal receptors (alpha-estrogen, beta-estrogen, progesteron), Ki-67 and P53 in endometrium of tamoxifen treated breast cancer patients. Rev. Med. Chir. Soc. Med. Nat. Iasi.

[45] Kübler K., Nardone A., Anand S., Gurevich D., Gao J., Droog M., Hermida-Prado F., Akhshi T., Feiglin A., Feit A.S. (2025). Tamoxifen induces PI3K activation in uterine cancer. Nat. Genet..

[46] Saeki H., Horimoto Y., Hlaing M.T., Men Y., Rong L., Ishizuka Y., Uomori T., Yoshida E., Terao Y., Arakawa A. (2024). Clinicopathological and molecular pathological characteristics in tamoxifen-related endometrial cancer. Oncol. Lett..

[47] Nagy E., Gajjar K.B., Patel I.I., Taylor S., Martin-Hirsch P.L., Stringfellow H.F., Martin F.L., Phillips D.H. (2014). MGMT promoter hypermethylation and K-RAS, PTEN and TP53 mutations in tamoxifen-exposed and non-exposed endometrial cancer cases. Br. J. Cancer.

[48] Turbiner J., Moreno-Bueno G., Dahiya S., Sánchez-Estevez C., Hardisson D., Prat J., Oliva E., Palacios J. (2008). Clinicopathological and molecular analysis of endometrial carcinoma associated with tamoxifen. Mod. Pathol..

[49] Droog M., Nevedomskaya E., Kim Y., Severson T., Flach K.D., Opdam M., Schuurman K., Gradowska P., Hauptmann M., Dackus G. (2016). Comparative Cistromics Reveals Genomic Cross-talk between FOXA1 and ERα in Tamoxifen-Associated Endometrial Carcinomas. Cancer Res..

[50] Hague S., Manek S., Oehler M.K., MacKenzie I.Z., Bicknell R., Rees M.C. (2002). Tamoxifen induction of angiogenic factor expression in endometrium. Br. J. Cancer.

[51] Dubé V., Grigull J., DeSouza L.V., Ghanny S., Colgan T.J., Romaschin A.D., Siu K.W. (2007). Verification of endometrial tissue biomarkers previously discovered using mass spectrometry-based proteomics by means of immunohistochemistry in a tissue microarray format. J. Proteome Res..

[52] Jin Q., Jiang X., Du X., Hu W., Bai S., Wang X., Xu B., Zhao W. (2021). Integrated Transcriptome and Multiple Activated Pathways in Endometrial Cancer. Front. Genet..

[53] Cao S., Chen N., Ma Y. (2025). Machine learning Nomogram for Predicting endometrial lesions after tamoxifen therapy in breast Cancer patients. Sci. Rep..

[54] Li C., Wang J., Zhang Y., Hu L., Wang Y. (2026). A machine learning-based risk prediction framework for atypical hyperplasia and endometrial cancer in postmenopausal women. World J. Surg. Oncol..

[55] Xu Q., Wu X., Guo Y. (2025). A high-risk prediction model for endometrial cancer: Exploring the synergistic interaction between polycystic ovary syndrome and metabolic syndrome. Am. J. Cancer Res..

[56] Ai F., Zhu H., Wang C., Zhou L., Wang S. (2025). A nomogram for the prediction of endometrial cancer in postmenopausal women with a thickened endometrium. BMC Womens Health.

[57] Collins G.S., Moons K.G.M., Dhiman P., Riley R.D., Beam A.L., Van Calster B., Ghassemi M., Liu X., Reitsma J.B., van Smeden M. (2024). TRIPOD+AI statement: Updated guidance for reporting clinical prediction models that use regression or machine learning methods. BMJ.

[58] Bossuyt P.M., Reitsma J.B., Bruns D.E., Gatsonis C.A., Glasziou P.P., Irwig L., Lijmer J.G., Moher D., Rennie D., de Vet H.C. (2015). STARD 2015: An Updated List of Essential Items for Reporting Diagnostic Accuracy Studies. Radiology.

[59] Sarioglu E., Vural F., Ertürk Coşkun A.D. (2023). The relationship of endometrial pathologies with endometrial thickness and inflammatory markers in breast cancers using tamoxifen. Arch. Gynecol. Obstet..

