# Supplementary material for: Diagnostic Assessment of Tamoxifen-Associated Endometrial Pathology: A Narrative Review of Biomarkers, Risk Models, and Translational Gaps
Source: Diagnostics (Basel). 2026 Oct 18;16(20):3388. doi: 10.3390/diagnostics16203388 (PMC13703038; doi:10.3390/diagnostics16203388)
Supplement: Supplementary file 1 [file diagnostics-16-03388-s001.zip › diagnostics-4542497-supplementary.pdf]

Supplementary Files

Supplementary Material S1

Search documentation

The original manuscript reported MEDLINE/PubMed searching and backward reference checking through August 2026. Its executable original queries, exact lower date boundary, language filters, and record-level screening decisions were unavailable in the revision documentation; the number of original search results, exclusions, and included studies is therefore not asserted.

The publication interval was 1 January 2000 to 31 August 2026, preserving the original upper cutoff; no language, study-design, or human-only interface filter was applied to this supplementary query.

Exact query

(tamoxifen[Title/Abstract] OR tamoxifen[MeSH Terms]) AND (endometrial[Title/Abstract] OR endometrium[MeSH Terms] OR uterine[Title/Abstract]) AND (diagnos*[Title/Abstract] OR ultrasound[Title/Abstract] OR ultrasonograph*[Title/Abstract] OR hysteroscop*[Title/Abstract] OR biops*[Title/Abstract] OR biomarker*[Title/Abstract] OR methylation[Title/Abstract] OR genomic*[Title/Abstract] OR predict*[Title/Abstract]) AND ("2000/01/01"[Date - Publication] : "2026/08/31"[Date - Publication])

Retrieved record count: 986. This is the count returned by the database query, not a screened, eligible, or included-study count; exhaustive screening of those records was not conducted. Source-specific searches by title, identifier, and author were used to check the studies underpinning revised claims, with accessible publisher full texts consulted for disputed metrics and validation designs.

The review uses human tamoxifen-exposed cohorts for direct clinical evidence, general human endometrial-disease studies as external comparators, and human tissue or translational studies for mechanistic context. No minimum sample size was imposed; small studies are explicitly identified as discovery or limited evidence. English reports or available English abstracts were used, and conference-only, unrelated breast-cancer outcome, and isolated cell-culture evidence was not used to support diagnostic performance. These are the principles applied to the revised narrative synthesis, not a retrospectively claimed registered screening protocol.

SANRA domain mapping

Importance: The introduction identifies the diagnostic difficulty caused by common benign tamoxifen-related changes and less frequent clinically significant disease.

Aims: The introduction states the clinical questions and separates present assessment from investigational detection and surveillance.

Search description: Section 2.1 and this supplement report the known original methods, missing documentation, and the exact revision query with its scope and limits.

Referencing: Numerical claims are linked to their source populations, source mismatches were corrected, and references follow first appearance.

Scientific reasoning: Sections 3.4 and 6.4 and Tables 1–3 distinguish applicability, endpoints, validation designs, and limitations; there is no pooled ranking.

Outcome data: Tables 2–3 present relevant metrics with explicit populations and endpoints, and Figure 1 labels denominators and its composite outcome.

This mapping uses SANRA as a reporting-quality aid [6]. No numerical SANRA score or independent quality certification is claimed, and the mapping is not a substitute for QUADAS-2 or PROBAST assessment of primary studies [7, 8].

Interpretation of the qualitative appraisal

Tables 2 and 3 in the manuscript identify study-level concerns for the principal diagnostic and prediction studies. Supplementary Table S1 extends this approach to tissue and molecular reports. The comments are qualitative interpretive limitations, not formal risk-of-bias grades; a concern not listed should not be assumed absent. In the source prediction reports, the later validation cohorts of Xu and Ai were from their respective development institutions, so these designs are described as temporal rather than geographically independent validation [55, 56].

Supplementary Table S1. Qualitative appraisal of selected tissue and molecular studies.

Study	Population and applicability	Target or assay	Specific design concern	Consequence for interpretation
Leão et al. [39]	Direct; 84 exposed benign polyps, 252 nonuser polyps, 84 atrophic controls	ER/PR immunohistochemistry; benign tissue comparisons	Cross-sectional surgical selection; no malignant target group or diagnostic threshold	Exposure-related PR differences do not classify EIN/carcinoma
Tregón et al. [40]	Contextual human exposure; 40 postmenopausal prolapse patients, 20 exposed for 21 days	Receptor and proliferation response	Short experimental exposure; not a breast-cancer diagnostic cohort	Informs biological response, not clinical cancer detection
Altaner et al. [41]	Direct tissue comparison; 14 exposed polyps with small control, hyperplasia, and cancer groups	Ki-67/Bcl-2 staining	Retrospective tissue selection; no locked cut-off or external validation	Higher proliferation does not establish future malignant transformation
Dibi et al. [42]	Direct; 14 exposed and 32 unexposed women with postmenopausal bleeding and polyps	ER, PR, Ki-67, Bcl-2	Small selected benign-lesion groups; no significant between-group differences	Contradicts a simple exposure-specific receptor/proliferation rule
Pokharel et al. [43]	Direct; 50 premenopausal/postmenopausal users treated for over two years	Serum p53 antibody and tissue p53 against histopathology	Small cohort; verification procedures depended on ultrasound; no independent validation	Serum sensitivity 50.0%; tissue sensitivity 57.1%; not stand-alone screening
Negoiţă and Mihailovici [44]	Direct; 20 symptomatic proliferative specimens selected from 125 patients	Receptor, Ki-67, and p53 patterns	Highly selected small tissue series; no validated classifier	Benign receptor positivity must not be described as universal receptor absence
Kübler et al. [45]	Direct tumor profiling with additional clinical and experimental datasets	PI3K pathway and tumor-genomic findings	Established cancers; experimental support does not constitute a human diagnostic validation	Fewer activating mutations can coexist with non-genetic pathway activation

Study	Population and applicability	Target or assay	Specific design concern	Consequence for interpretation
Saeki et al. [46]	Direct; 10 exposed and 20 sporadic cancers	Copy number, MSI, and MMR immunohistochemistry	Very small exposed group; no representative benign exposed control series	Four MSI-high exposed cancers do not define a screening test
Nagy et al. [47]	Direct; 30 exposed and 38 unexposed cancers	MGMT methylation and mutation profiles	Tumor-grade imbalance; modest sample; co-occurrence difference not statistically significant	78% versus 50% concerns methylation–mutation co-occurrence, not overall methylation prevalence
Turbiner et al. [48]	Direct; 18 exposed cancers and selected comparator groups	β-Catenin, KRAS, PTEN, and MSI	Smaller informative subsets; variable tumor comparators; several nonsignificant comparisons	Hypothesis-generating tumor differences cannot be assembled into a validated signature
Droog et al. [49]	Direct tumor component with experimental analyses; 230 tumor specimens for immunohistochemistry	ERα/FOXA1 cistromics and interval to cancer	Retrospective tumor selection; temporal association is not diagnostic accuracy	No demonstrated minimally invasive detection performance
Hague et al. [50]	Direct tissue biology; exposed and unexposed pre/postmenopausal endometrium	Angiogenic protein and vascular changes	Menopausal heterogeneity; exposure-response endpoint; no clinical threshold	Angiogenesis can reflect exposure without identifying malignant disease
Dubé et al. [51]	External; 148 tissue-microarray samples	Three-protein panel for malignant versus benign tissue	Internal random-split validation; resected tissue; no tamoxifen subgroup	Tissue performance cannot be assigned to a blood or urine assay
Jin et al. [52]	External; five paired cancer/normal tissues, with additional tissue RT-qPCR	Differential gene expression and pathway analysis	Very small discovery set; no diagnostic classifier or independent biofluid validation	Molecular discovery remains several steps from clinical triage
